# Regulation of Leukemic Cell Differentiation through the Vitamin D Receptor at the Levels of Intracellular Signal Transduction, Gene Transcription, and Protein Trafficking and Stability

**DOI:** 10.1155/2012/713243

**Published:** 2012-05-14

**Authors:** Elżbieta Gocek, Hanna Baurska, Aleksandra Marchwicka, Ewa Marcinkowska

**Affiliations:** Faculty of Biotechnology, University of Wrocław, Tamka 2, 50-137 Wrocław, Poland

## Abstract

1**α**,25-Dihydroxyvitamin D_3_ (1,25(OH)_2_D) exerts its biological activities through vitamin D receptor (VDR), which is a member of the superfamily of steroid receptors, that act as ligand-dependent transcription factors. Ligated VDR in complex with retinoid X receptor (RXR) binds to regulatory regions of 1,25(OH)_2_D-target genes. 1,25(OH)_2_D is able to induce differentiation of leukemic blasts towards macrophage-like cells. Many different acute myeloid leukemia (AML) cell lines respond to 1,25(OH)_2_D by increasing CD14 cell surface receptor, some additionally upregulate CD11b and CD11c integrins. In untreated AML cells VDR protein is present in cytosol at a very low level, even though its mRNA is continuously expressed. Ligation of VDR causes protein stabilization and translocation to the cell nuclei, where it regulates transcription of target genes. Several important groups of genes are regulated by 1,25(OH)_2_D in HL60 cells. These genes include differentiation-related genes involved in macrophage function, as well as a gene regulating degradation of 1,25(OH)_2_D, namely CYP24A1. We summarize here the data which demonstrate that though some cellular responses to 1,25(OH)_2_D in AML cells are transcription-dependent, there are many others which depend on intracellular signal transduction, protein trafficking and stabilization. The final effect of 1,25(OH)_2_D action in leukemic cells requires all these acting together.

## 1. Introduction

The primary role of 1*α*,25-dihydroxyvitamin D_3_ (1,25(OH)_2_D) is to maintain calcium and phosphate homeostasis in vertebrate organisms via actions in the intestine, bones, kidneys, and parathyroid glands. However, it is well known that physiological roles of 1,25(OH)_2_D reach much beyond calcium and phosphate homeostasis. For example, 1,25(OH)_2_D induces differentiation and inhibits proliferation of various normal and cancer cells, including osteoclasts, keratinocytes, and monocytes. In 1981 the group of Suda observed that 1,25(OH)_2_D was able to induce differentiation in the M1 murine myeloid cell line [1] and that it extended the survival of mice inoculated with leukemia cells [2]. Since then many research projects have been performed in order to prepare ground for clinical use of 1,25(OH)_2_D or of its low calcemic analogs in leukemia treatment [3–5].

There are two major signal transduction pathways activated by 1,25(OH)_2_D in target cells. The most important and the best documented is the so-called “genomic pathway,” with its most important player a vitamin D receptor (VDR). The less well described is “nongenomic pathway,” which consists of intracellular signalling molecules, such as mitogen-activated protein kinases (MAPKs), phosphatidylinositol 3-kinase (PI3K), and others, activated by mechanisms that are not fully understood now [6]. It is believed that both pathways need to be activated for full biological activity of 1,25(OH)_2_D and that the most probable mediator of these actions is a putative membrane VDR (mVDR) [6].

VDR belongs to the superfamily of intracellular receptors for steroid and thyroid hormones. 48 members of the superfamily have been identified in humans; they act as ligand-induced transcription factors [7]. Most of the superfamily members, in order to be biologically active, form homo- or heterodimers. For VDR, retinoid X receptor (RXR) is a dimerization partner. VDR upon ligation undergoes conformational changes that allow binding to specific sequences in promoter regions of target genes, called vitamin D response elements (VDREs). VDREs are composed of two repeated half-sites with the consensus sequence AGGTCAcagAGGTCA (VDRE-DR3). Binding of 1,25(OH)_2_D to VDR enhances heterodimerization with RXR and allows binding of the coactivator complex, known as vitamin D receptor-interacting protein complex (DRIP) [8] and of other proteins, histone acetylase among them. Acetylated histones relax chromatin structure to make DNA accessible and permit initiation of transcription of target genes [9]. VDR may be ligated not only with 1,25(OH)_2_D but also with other compounds such as lithocholic acid, docosahexaenoic acid, arachidonic acid, or curcumin [10]. Moreover, there are about 300 compounds closely related to 1,25(OH)_2_D, called 1,25(OH)_2_D analogs, which can bind VDR and exert changed biological properties. Subtle conformational changes in VDR structure caused by analogs can produce antagonistic, agonistic, or even superagonistic effects. There are even some analogs that exert semiselective activities, with lowered calcemic and increased antiproliferative and cell differentiating effects [11].

The VDR protein is expressed at low concentrations in target tissues and cultured cells with the level of receptor expression ranging from a few copies of the VDR/cell to 25 000 copies/cell [7]. Among blood cells VDR is expressed in Tcells, Bcells, monocytes, neutrophils, platelets, macrophages, and dendritic cells. Also many different myeloid leukemia cell lines, blocked at various stages of maturation, expressed mRNA for VDR; however, the expression levels were variable [12]. Addition of 1,25(OH)_2_D to certain acute myeloid leukemia (AML) cells induces dramatic changes in their phenotype and function; however, the extent of these changes is various in various cell lines.

The activation of MAPK/Erk1,2 signal transduction pathway in AML cells in response to 1,25(OH)_2_D was for the first time reported in 1997 [13], and it was later shown to be important for the process of AML cell differentiation [14]. The exact mechanisms of how MAPK/Erk1,2 participate in the differentiation process are not known; however, they are being connected with a proliferative phase of AML cells differentiation [15]. MAPK/JNK pathway, whose activation was reported later [16], appeared to be involved in a subtle way in regulation of 1,25(OH)_2_D-dependent transcription factors [17]. Another MAP kinase, p38, has antagonistic effects to both MAPK/Erk1,2 and MAPK/JNK [16, 18]. Also activation of PI3K signal transduction pathway in AML cells exposed to 1,25(OH)_2_D has been reported [19] and was later shown to be responsible for activation of myeloid zinc finger-1 (MZF-1) transcription factor, which in turn participates in regulation of proteins crucial for macrophage function [20].

## 2. Nuclear Trafficking of VDR

VDR to fulfil its function of nuclear receptor must be transported into the nucleus [21]. In eukaryotic cells nucleoplasm and genetic material are separated from the cytosol by a double membrane which contains highly selective, bidirectional transporter channel called nuclear pore complex (NPC) [22]. NPC is composed of nearly 30 proteins termed nuclear pore complex components or “nucleoporins” (NUPs) [22] which occur in multiple copies [23]. Nuclear import of proteins through NPC is mediated by transporter proteins, such as importin*α* and importin*β*, which bind cargoes through nuclear localisation signal (NLS) and interact with NUPs. Complex cargo-importin*α* binds importin*β* which interacts with Ran-GDP protein [21]. Ran-GDP exists mostly in cytoplasm, whereas Ran-GTP in nuclei and this GTP gradient ensures the right direction of nuclear transport [21]. High concentration of Ran-GDP promotes the formation of import complex, while high concentration of Ran-GTP dissociates them and promotes formation of export complexes [24]. To complete the cycle importin*α* and importin*β* must be transported back to the cytoplasm. To ensure Ran-GTP gradient, in the cytosol Ran-guanosine triphosphatase activating protein (RanGAP) stimulates the intrinsic GTP-hydrolyzing activity of Ran to form Ran-GDP. Hydrolysis of Ran-GTP to Ran-GDP causes release of importin*β* for the next cycle.

**Figure 1:**
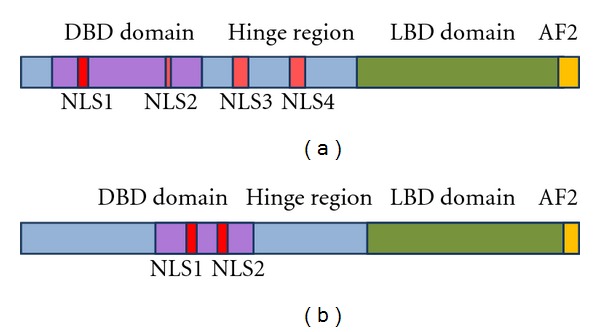
Localization of nuclear import domains (NLS) in VDR and RXR. NLS segments in VDR (a) and in RXR (b). Based on [26].

**Figure 2:**
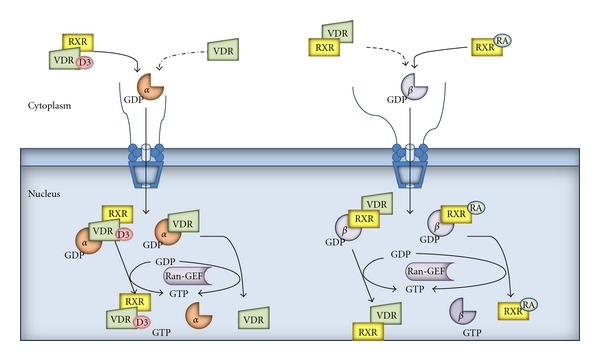
Nuclear import of VDR, RXR, and their heterodimer. GTP: guanosine 5′-triphosphate, GDP: guanosine diphosphate, *α*: importin*α*, *β*: importin*β*, and RA: retinoic acid. Based on [27, 28].

To overcome the NPC, large molecules, such as nuclear receptors, harbour NLSs recognized by the transporter proteins which interact with NUPs in NPC. In VDR four NLSs were indentified (Figure 1(a)). First (NLS1) is localized between two zinc fingers within DNA-binding domain (DBD) [29]. The second NLS is in the second zinc finger of the DBD but data show that NLS2 does not function as an obligatory NLS. The next two are localized in the hinge region of VDR. NLS3 (102–110) is important for ligand-induced nuclear localisation of VDR but has no effect on unligated VDR nuclear import. NLS4 (154–173) is a short segment without a confirmed function. The RXR which is a partner protein for VDR has two NLSs, the first localized between two zinc fingers in DBD and the second in the second zinc finger (Figure 1(b)) [26]. Some data demonstrated that VDR shuttles between nucleus and cytoplasm in the absence of ligand, but unligated VDR weakly interacts with importin*α* [27]. However, nuclear trafficking of unligated VDR involves importin 4 through the interaction with the aminoterminus of VDR [21]. Binding the ligand promotes heterodimerization with RXR and enhances nuclear localization. Whereas RXR is predominantly localized in the nuclei even in the absence of ligand, there is also a cytoplasmic fraction of RXR that is translocated to the nucleus in the presence of its ligand. The nuclear import of RXR is mediated through importin*β*, while that of VDR is mediated through importin*α* (Figure 2) [27]. In their publication Prüfer and Barsony showed that VDR is imported using two different pathways: ligand-dependent and ligand-independent. In ligand independent pathway RXR is the dominant partner for nuclear translocation, whereas ligated VDR dominates the mobility of RXR [28]. Binding 1,25(OH)_2_D stabilizes heterodimer VDR : RXR and its interaction with importin*α* but inhibits interaction of RXR with importin*β* [27].

In order to let proteins out of the nucleus, exportin named the chromosomal region maintenance 1 protein (CRM1) recognizes proteins containing leucine-rich nuclear export signals (NESs). Export complex formation is favored by high concentrations of RanGTP in the nucleus, which facilitate specific interactions with nucleoporins at the nuclear basket for appropriate translocation through the NPC [25]. VDR utilizes two pathways of nuclear export: ligand dependent and ligand independent. Unligated VDR uses a CRM1 export mechanism using NES localized in position 320–325 in LBD [28]. Mechanism for liganded VDR is CRM1 indepentent and requires DBD which functions as NES [30].

## 3. Differentiation of Human AML Cell Lines in Response to 1,25(OH)_**2**_D

**Figure 3:**
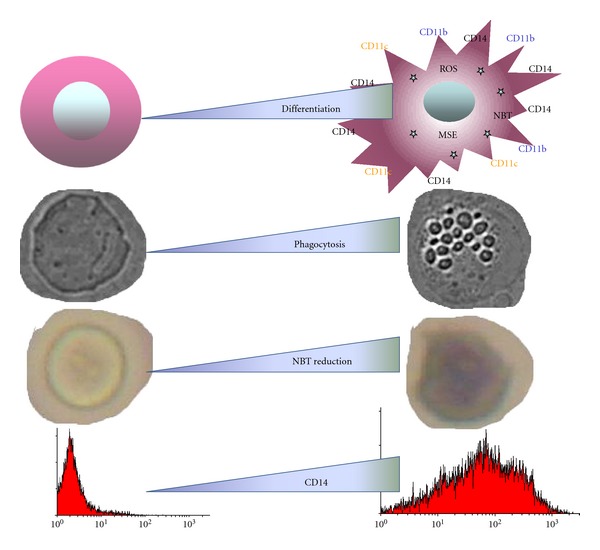
1,25(OH)_2_D-induced changes in AML cells. MSE: monocyte specyfic esterase; ROS: reactive oxygen species; NBT: nitroblue tetrazolium, CD14: co-receptor for LPS, CD11b: subunit of *α*_M_ *β*2 integrin, CD11c: integrin *α*X. Based on [31].

 One of the major processes in the array of anticancer actions of 1,25(OH)_2_D is differentiation of AML cells. Differentiation in AML cells consists of a G0/G1 cell cycle block [32], which is connected to an increase of proteins p21 and p27 [33], an increase in expression of antiapoptotic proteins [34], and acquisition of functional and phenotypic features characteristic for normal macrophages. Functional features are connected with an ability to phagocytose [35], with increased activity of monocyte specific esterase (MSE) [36], and with an ability to generate reactive oxygen species (ROS) and reduce nitroblue tetrazolium (NBT) [37]. Differentiation is also accompanied by upregulation of certain cell surface molecules, which are necessary for macrophage function, such as CD14, which is a coreceptor for lipopolysaccharide (LPS), as well as CD11b, which is a subunit of *α*_M_ *β*_2_ integrin or CD11c, an integrin *α*X, both involved in the cell adhesion [31, 38]. The process of 1,25(OH)_2_D-induced AML cell differentiation is not fast, it requires 3-4 days to reach plateau in expression of cell surface antigens, but differences in cell phenotype and function are spectacular (Figure 3). Since differentiation of blood cells may have beneficial effects, therapeutic applications for 1,25(OH)_2_D have been postulated. However, a major limitation for therapeutic use of 1,25(OH)_2_D is its potent calcemic and phosphatemic activity. The doses of 1,25(OH)_2_D, which are necessary to inhibit cell proliferation and induce differentiation, produce in vivo hypercalcemia and hyperphosphatemia that may be life threatening. Therefore there is a need for new 1,25(OH)_2_D analogs that retain high differentiating and antiproliferative activities with minimal or tolerable calcemic and phosphatemic effects [39]. Many such analogs were tested in our laboratory and their activities in inducing differentiation of various AML cell lines, as well as differentiation of leukemic blasts from the peripheral blood of AML patients, were studied.

## 4. Side-Chain Modified Analogs of Vitamin D_**2**_ and Vitamin D_**3**_

In our previous papers we described prodifferentiating activities of various side-chain modified analogs of vitamin D_3_, PRI-2191, PRI-2201, PRI-2202, and PRI-2205 and of vitamin D_2_, PRI-1906, PRI-1907 PRI-1908, and PRI-1909 [40, 41, 45–48]. We studied their pro-differentiating activities towards various human AML cell lines with genetic lesions characteristic for AML. Our studies indicated that some of the tested analogs were more potent than 1,25(OH)_2_D in induction of cell differentiation. The most efficient were the two analogs of vitamin D_3_, named PRI-2191 and PRI-2201, and the two analogs of vitamin D_2_, PRI-1906 and PRI-1907 [40–42]. Interestingly, one of the most active analogs tested by our group was PRI-2191, which in fact is a 1,25(OH)_2_D metabolite, already in use for treatment of psoriasis [41, 49] and a potential drug for vitiligo [50]. The four analogs mentioned above were tested in vivo for their calcemic activity. Results of these studies showed that PRI-2191, PRI-2201, and PRI-1906 were less calcemic than 1,25(OH)_2_D, while PRI-1907 was comparable to 1,25(OH)_2_D [48, 51, 52].

**Figure 4:**
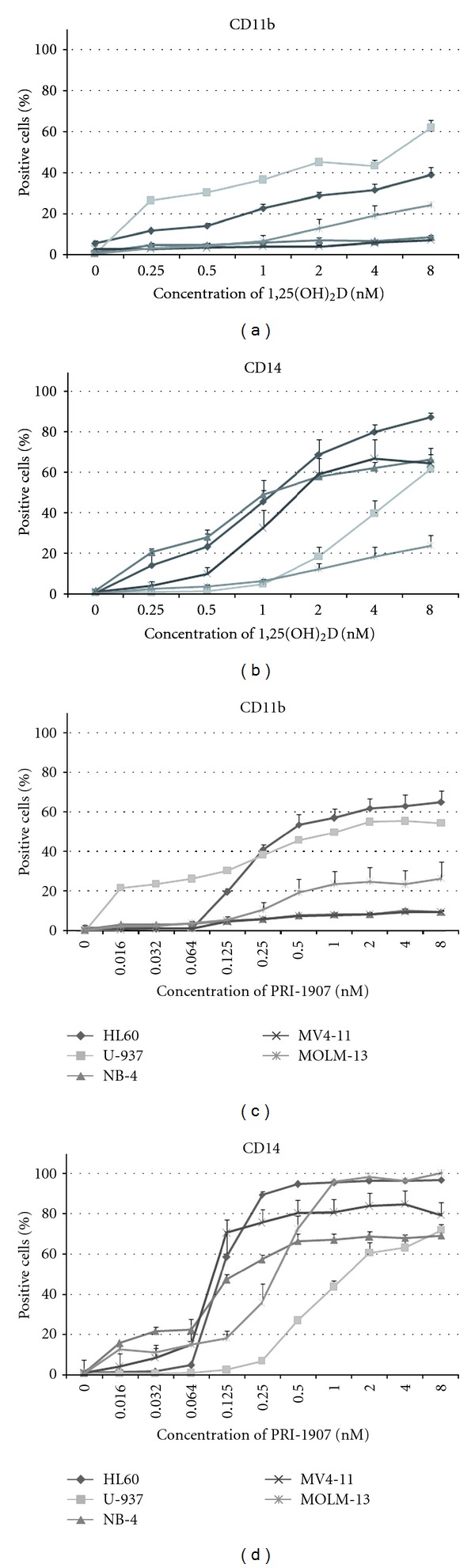
Expression of monocytic differentiation markers in AML cell lines exposed to either 1,25(OH)_2_D or to PRI-1907. HL60, U-937, NB-4, MV4-11, and MOLM-13 cells were exposed either to 1,25(OH)_2_D (a, b) or to PRI-1907 (c, d) for 96 h, and then the expression of CD11b and CD14 was tested in flow cytometry. The graphs show mean percentages (±SEM) of cells expressing cell differentiation markers. Based on [40].

The cell lines used in our studies were derived from various AML subtypes. HL60 cells originate from M2 subtype of AML and are the most sensitive to 1,25(OH)_2_D-induced differentiation out of all cell lines used in our tests. NB-4 cells carry the t(15;17) PML-RARA fusion gene, which is characteristic for AML M3, U937 cells carry translocation t(10;11) often seen in AML M5, MV4-11 cells express fusion gene MLL-AF4, and MOLM13 cells express fusion gene MLL-AF9. Moreover, MOLM13 and MV4-11 have an internal tandem duplication in Flt3 gene (Flt3-ITD), in one or in both alleles, respectively [53]. Our studies revealed that in all cell lines studied, 1,25(OH)_2_D and its active analogs were able to upregulate CD14 cell surface antigen, while only in some cell lines CD11b integrin level was elevated (Figure 4). These data demonstrate that the expressions of CD14 and CD11b are controlled by two different signal transduction pathways, and in some AML cells CD11b pathway is blocked and cannot be overcome by 1,25(OH)_2_D.

## 5. VDR in Human AML Cell Lines

**Figure 5:**
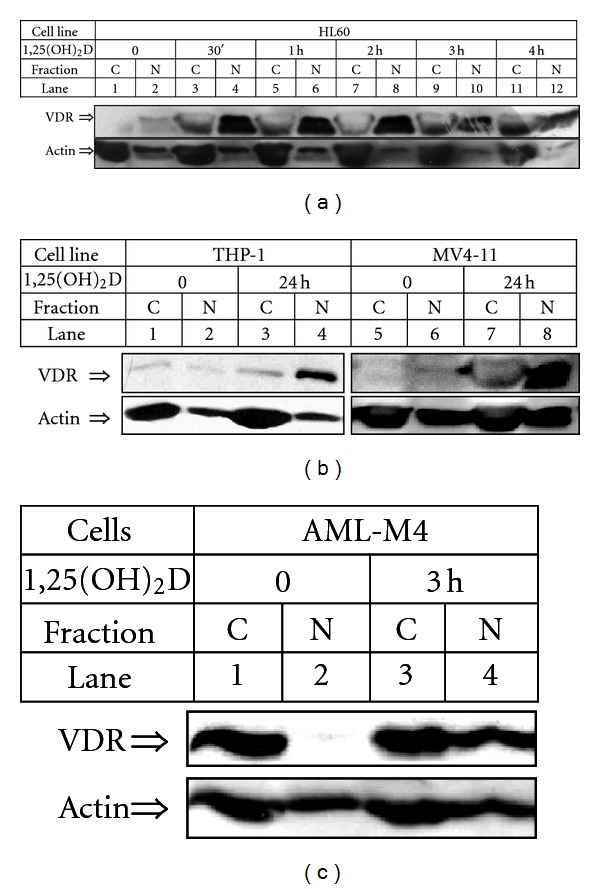
Expression of VDR protein in AML cells exposed to 1,25(OH)_2_D. AML cells after incubation for indicated times with 10 nM 1,25(OH)_2_D were lysed and fractionated into the cytoplasmic (C) and nuclear (N) fractions. The lysates from equal numbers of cells were separated in SDS-PAGE and blotted to the membrane. The membrane was probed with anti-VDR. Actin was probed as a control of equal loading and transfer of proteins. (a) HL60 cells, (b) THP-1, MV4-11 cells, and (c) AML-M4 blasts from patient's peripheral blood. Based on [41, 42] and on unpublished data.

**Figure 6:**
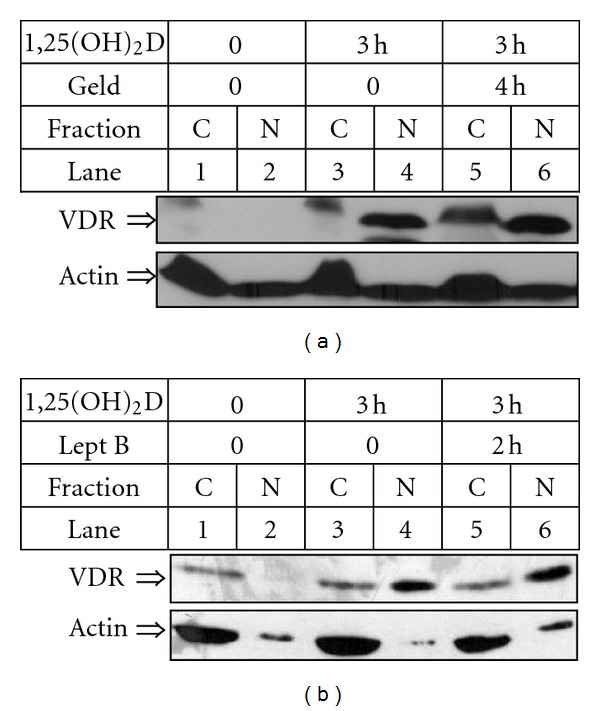
Nuclear trafficking of VDR in presence of geldanamycin (a) or leptomycin B (b). HL60 cells were exposed to 10 nM 1,25(OH)_2_D for 3 h. One sample was pretreated with 1 *μ*M geldanamycin (geld) for 1 h before exposure to 1,25(OH)_2_D (a). Another sample was treated with 5 mg/ml of leptomycin B (lept B) for the last two hours of incubation (b). After incubation the cells were lysed and fractionated into the cytoplasmic (C) and nuclear (N) fractions. The lysates from equal numbers of cells were separated in SDS-PAGE and blotted to the membrane. The membrane was probed with anti-VDR. Actin was probed as a control of equal loading and transfer of proteins.

Experiments made by our group showed that AML cell lines have very low constitutive level of VDR protein, which increases significantly after exposure of the cells to 1,25(OH)_2_D. Therefore if the proposed nuclear import and export of unligated VDR exists in AML cells, it remains at a very low level, which is difficult to detect. 1,25(OH)_2_D-induced changes in nuclear trafficking of VDR could be observed using confocal microscopy, but western blotting of cell lysates fractionated into cytosol and nuclei appeared to be a much more sensitive method of VDR detection [54]. The kinetics of VDR accumulation in AML cells is surprisingly fast after exposure of the cells to 1,25(OH)_2_D. In HL60 cells, VDR starts to accumulate in the cell nuclei after few minutes from exposure to 1,25(OH)_2_D [42] and after half of an hour the difference is significant (Figure 5(a)). However, most of our studies were performed using HL60 cell line; also in other cell lines (Figure 5(b)) and in AML blasts (Figure 5(c)) accumulation of VDR in cell nuclei was observed [42, 54]. Obviously, the increase has not been caused through transcription of new mRNA for VDR since mRNA levels remained almost unchanged by 1,25(OH)_2_D, as confirmed by real-time PCR (not shown). Therefore the mechanism of accumulation of VDR must be regulated at post transcriptional level, and according to our data it is caused by ligation-induced protection of VDR protein from degradation [42]. It seems that VDR is continuously produced in the cytosol, and as long as it is unligated, it undergoes degradation. Ligation of VDR with 1,25(OH)_2_D induces rapid translocation of the receptor to the cell nuclei, where degradation process is slower. For effective nuclear trafficking, active p42,44/MAPK pathway and active PI3-K pathway are needed; however, the mechanism of this effect is not known [54]. It was surprising that in most of freshly isolated AML cells from patients constitutive level of VDR in cytosol was higher than in established cell lines [41]. Unfortunately we were unable to test this phenomenon in a more detailed manner, because neither of the patient's samples was possible to be cultured in vitro for longer than two-three weeks. It is known; however, that other nuclear receptors become stabilized in the cytosol by heat shock proteins (Hsp) [55, 56], so the involvement of this class of proteins was addressed. We demonstrated recently that, in HL60 cells, VDR interacts with Hsp90 and that activation of Hsp90 is necessary for the differentiation process [57], but our new experiments documented that activation of Hsp90 is not necessary for nuclear translocation of VDR. Geldanamycin, which inhibits activity of Hsp90, was not able to block 1,25(OH)_2_D-induced nuclear accumulation of VDR (Figure 6(a)). VDR protein appears in the nuclei of 1,25(OH)_2_D-treated cells very fast but disappears slowly. Our experiments in which HL60 cells were exposed to 1,25(OH)_2_D and an inhibitor of CRM1, namely, leptomycin B, confirmed that nuclear export of ligated VDR is CRM1 independent. As presented in Figure 6(b), presence of leptomycin B did not cause further accumulation of VDR in cells exposed to 1,25(OH)_2_D. As it was presented before, VDR protein levels remain elevated even after 4 days from exposure of HL60 cells to 1,25(OH)_2_D [42]. During this time VDR activates transcription of its target genes. One of them is CYP24A1, which encodes an enzyme, 24-hydroxylase of 1,25(OH)_2_D, responsible for degradation of 1,25(OH)_2_D to calcitrioic acid. As presented in our previous publication, 1,25(OH)_2_D increased in HL60 cells levels of CYP24A1 mRNA significantly; however, kinetics of induction was very slow [40]. CYP24A1 protein is localized exclusively in an inner membrane of mitochondria, where its levels also increase slowly after exposure to 1,25(OH)_2_D (not shown here).

## 6. Regulation of C/EBP Transcription Factors by 1,25(OH)_**2**_D in AML Cells

**Figure 7:**
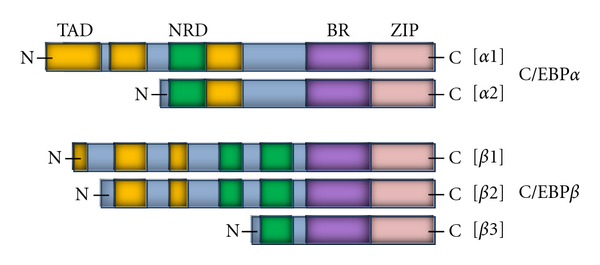
Schematic representation of the C/EBP*α* and C/EBP*β* isoforms. TAD: transcription activation domain, NRD: negative regulatory domain, BR: basic region, and ZIP: leucine zipper. Based on [43, 44].

There are several important groups of genes regulated by 1,25(OH)_2_D in AML cells, including differentiation-related genes that encode proteins important for function of monocytes or macrophages. The examples of such are genes encoding CAAT-enhancer binding proteins (C/EBPs), belonging to the family of basic leucine zipper (bZIP) transcription factors [44, 58, 59]. There are six genes for different C/EBPs (*α*, *β*, *γ*, *δ*, *ε*, and *ζ*) which are expressed in hematopoietic cells, hepatocytes, adipocytes, spleen, kidney, brain, and others. They can form homodimers and heterodimers with other family members and with other transcription factors. The C/EBP proteins contain highly conserved bZIP domain at the C-terminus, an activation domain at the N-terminus and some other regulatory domains (Figure 7) [43]. In hematopoietic cells C/EBP*α* is necessary mainly for differentiation from lymphoid-myeloid progenitors to granulocytes [60], while C/EBP*β* is crucial for specialization of normal and 1,25(OH)_2_D-induced monocytes and macrophages, as well as for their proper functions [61–64], as it regulates transcription of many monocyte-specific proteins, such as CD14, lactoferrin, or lysozyme [43]. Recently an involvement of C/EBP*β* in differentiation-related inhibition of proliferation was reported [65]. Because of alternative translation initiations sites, two different products of C/EBP*α* (42 kDa, 30 kDa) and three products of C/EBP*β* genes are translated (55 kDa, 49 kDa, 20 kDa) [43, 66]. As presented in Figure 7, 30 kDa form of C/EBP*α* and 20 kDa form of C/EBP*β* are devoid of portions of N-termini where transactivation domains are localized. They are, however, still able to dimerize, and therefore they can play an inhibitory function. Experiments done by groups of Studzinski and ours have shown that after exposure of HL60 to 1,25(OH)_2_D C/EBP*α* was only transiently upregulated in an early phase of differentiation, whilst upregulation of C/EBP*β* was strong, long-lasting and correlated with the differentiation process [67]. Specially two shorter C/EBP*β* isoforms were abundant in differentiating cells and their increase correlated with acquisition of monocytic differentiation markers, such as CD11b, CD14 [67], or CD11c (not shown).

**Figure 8:**
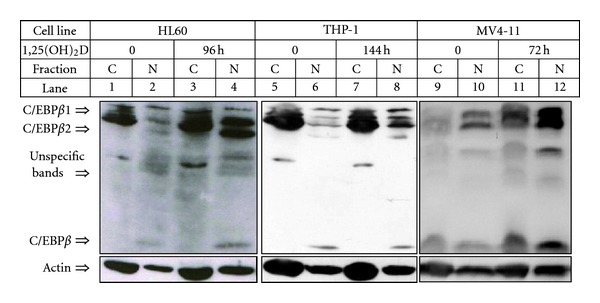
Subcellular localization of C/EBP*β* isoforms in AML cells. The cells were exposed to 10 nM 1,25(OH)_2_D for given times and then were fractionated into cytoplasmic (C) and nuclear (N) fractions. The lysates from equal numbers of cells were separated in SDS-PAGE and blotted to the membrane. The membranes were probed with anti-C/EBP*β* antibody. Actin was probed as a control of equal loading and transfer of proteins.

**Figure 9:**
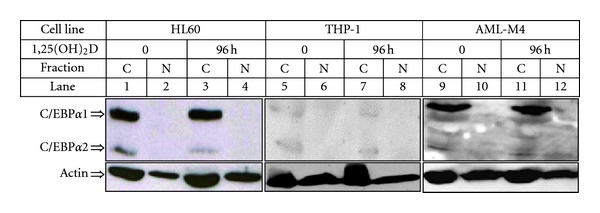
Subcellular localization of C/EBP*α* isoforms in AML cells. HL60 and AML-M4 blasts from patient's peripheral blood were exposed to 10 nM 1,25(OH)_2_D for 96 h and then were fractionated into cytoplasmic (C) and nuclear (N) fractions. The lysates from equal numbers of cells were separated in SDS-PAGE and blotted to the membrane. The membrane was probed with anti-C/EBP*α* antibody. Actin was probed as a control of equal loading and transfer of proteins.

Transcriptional activity of C/EBP proteins is regulated not only by their length and dimerization but most importantly by their intracellular localization. As transcription factors, C/EBPs must enter the cell nuclei to bind CCAAT box motif in their target gene promoters [44, 66]. Therefore, cellular trafficking of C/EBPs was extensively studied by our group. Again, studied cells were fractionated into cytosol and nuclei. As shown in Figure 8, after exposure of the cells to 1,25(OH)_2_D, the full length isoform of C/EBP*β*-1 (55 kDa) is present in either cytoplasmic or nuclear fraction, whilst the majority of shorter isoforms C/EBP*β*-2 (49 kDa) and C/EBP*β*-3 (20 kDa) are placed in the nuclei of the cells. Similar results were observed in various AML cell lines, such as THP-1, MV4-11, or MOLM-13 and in some samples of AML blasts isolated from patients. But when localization of C/EBP*α* was tested in fractionated cells, it appeared to be cytoplasmic, and no translocation was noticed after various times of exposure of the cells to 1,25(OH)_2_D. As examples, HL60, THP-1 cell lines, and AML blasts from patient's peripheral blood are presented (Figure 9). These findings suggest that, in AML cells, even if not mutated, C/EBP*α* is transcriptionally inactive what leads to the disturbances in granulopoiesis. Elevated expression of C/EBP*β* and its nuclear translocation induced by 1,25(OH)_2_D can possibly allow the cells to bypass this block and switch differentiation into monocyte/macrophage pathway. The above hypothesis was presented before [68] and is further supported by the findings shown here, that in AML cells C/EBP*α* is localized in the cytosol, where it cannot exert its transcriptional activity.

## 7. Conclusions

**Figure 10:**
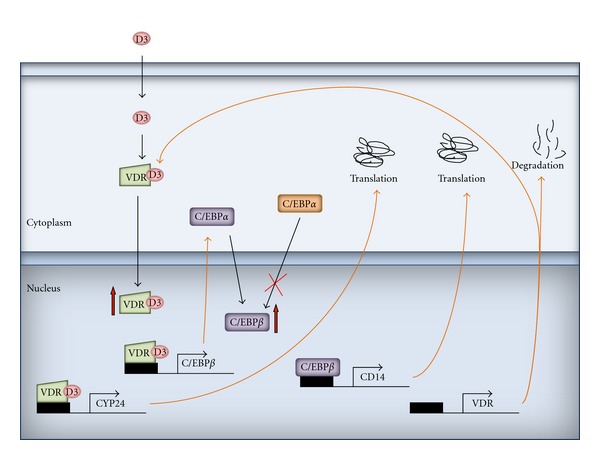
Overview of 1,25(OH)_2_D-induced intracellular events in AML cells. Description is in the text.

Exposure of AML cells to 1,25(OH)_2_D or to its analogs triggers a long series of events which eventually lead to acquisition of monocyte/macrophage phenotype and function (some of them presented in Figure 10). The detailed description of 1,25(OH)_2_D-induced differentiation in cell line models, as well as in AML blasts isolated from patients might be important for future therapeutic applications of 1,25(OH)_2_D analogs. It is especially significant to learn which differentiation pathways are blocked in certain AML subtypes and how they could be bypassed with help of pharmacological agents.
